# Neuropathologic basis of frontotemporal dementia in progressive supranuclear palsy

**DOI:** 10.1002/mds.27816

**Published:** 2019-08-21

**Authors:** Nobutaka Sakae, Keith A. Josephs, Irene Litvan, Melissa E. Murray, Ranjan Duara, Ryan J. Uitti, Zbigniew K. Wszolek, Neil R. Graff‐Radford, Dennis W. Dickson

**Affiliations:** ^1^ Department of Neuroscience Mayo Clinic Jacksonville, Florida USA; ^2^ Department of Neurology Mayo Clinic Rochester Minnesota USA; ^3^ Department of Neurology University of California San Diego La Jolla, California USA; ^4^ Mount Sinai Medical Center Miami Beach, Florida USA; ^5^ Department of Neurology Mayo Clinic Jacksonville, Florida USA

**Keywords:** behavioral variant frontotemporal dementia, immunohistochemistry, image analysis, progressive supranuclear palsy, tau

## Abstract

**Background:**

Progressive supranuclear palsy (PSP) is a neurodegenerative disorder characterized by neuronal loss in the extrapyramidal system with pathologic accumulation of tau in neurons and glia. The most common clinical presentation of PSP, referred to as Richardson syndrome, is that of atypical parkinsonism with vertical gaze palsy, axial rigidity, and frequent falls. Although cognitive deficits in PSP are often ascribed to subcortical dysfunction, a subset of patients has dementia with behavioral features similar to the behavioral variant of frontotemporal dementia. In this study we aimed to identify the clinical and pathological characteristics of PSP presenting with frontotemporal dementia.

**Methods:**

In this study, we compared clinical and pathologic characteristics of 31 patients with PSP with Richardson syndrome with 15 patients with PSP with frontotemporal dementia. For pathological analysis, we used semiquantitative methods to assess neuronal and glial lesions with tau immunohistochemistry, as well image analysis of tau burden using digital microscopic methods.

**Results:**

We found greater frontal and temporal neocortical neuronal tau pathology in PSP with frontotemporal dementia compared with PSP with Richardson syndrome. White matter tau pathology was also greater in PSP with frontotemporal dementia than PSP with Richardson syndrome. Genetic and demographic factors were not associated with atypical distribution of tau pathology in PSP with frontotemporal dementia.

**Conclusions:**

The results confirm the subset of cognitive‐predominant PSP mimicking frontotemporal dementia in PSP. PSP with frontotemporal dementia has distinct clinical features that differ from PSP with Richardson syndrome, as well as differences in distribution and density of tau pathology. © 2019 The Authors. *Movement Disorders* published by Wiley Periodicals, Inc. on behalf of International Parkinson and Movement Disorder Society.

Progressive supranuclear palsy (PSP) is a distinctive clinicopathologic disorder characterized by accumulation of insoluble microtubule‐associated tau protein enriched in the 4‐repeat isoform.^1^ Tau pathology is found in vulnerable regions of the neocortex and subcortical structures, and it takes the form of neurofibrillary tangles and pretangles in neurons, coiled bodies in oligodendroglia, and tufts in astrocytes (“tufted astrocytes”).^1^ The most common clinical presentation of PSP is akinetic‐rigid parkinsonism with vertical gaze palsy, axial rigidity, and frequent falls, referred to as Richardson syndrome (PSP‐RS).^2^ Atypical presentations of PSP are increasingly recognized, including parkinsonism (PSP‐P), pure akinesia with gait failure (PSP‐PAGF), progressive nonfluent aphasia, and corticobasal syndrome (PSP‐CBS).^1^ The latter 2 are associated with disproportionate neocortical involvement. Less frequently, PSP can present with clinical features similar to the behavioral variant frontotemporal dementia (PSP‐FTD).^3^, ^4^, ^5^ Although a number of studies have reported the correlation between the clinical phenotype and the distribution and severity of tau pathology in PSP‐RS, PSP‐P, PSP‐CBS, and PSP‐PAGF,^2^, ^6^, ^7^, ^8^ given the rarity of PSP‐FTD, there are few neuropathologic studies; most of the current literature includes case reports or small case series. Hassan and coworkers reported 3 patients with PSP‐FTD who had initial behavioral abnormalities, followed years later with motor symptoms suggestive of PSP.^4^ Respondek and coworkers described 12 patients with clinical features of FTD in a multicenter study of 100 autopsy‐confirmed PSP patients, but did not include details on the nature or distribution of tau pathology.^5^ Because the main clinical phenotype of FTD is behavioral change, we hypothesized that frontotemporal pathology would correlate with these symptoms. Therefore, we selected superior and middle frontal gyri and the inferior temporal gyrus as regions of interest. In the present study, we documented clinicopathological characteristics of PSP‐FTD compared with PSP‐RS and systematically assessed tau pathology in neocortical and subcortical regions, with attention to distribution and density, as well as its cellular characteristics.

## Materials and Methods

### Case Materials

Patients with a neuropathological diagnosis of progressive supranuclear palsy were identified from the neuropathology files of the Mayo Clinic Jacksonville brain bank between 1998 and 2017. Brain autopsies were obtained after consent of the next of kin or someone with legal authority to grant permission. Most brains were submitted as part of the CurePSP Brain Bank.^9^ The Mayo Clinic institutional review board has determined that research on postmortem brain samples is exempt from human subject research under HHS 45 CFR 46.101(b). Of 1229 cases with autopsy‐confirmed PSP in this period, 27 cases (2%) had prominent behavioral disturbance, and 15 cases met criteria for behavioral variant FTD (bvFTD). In 7 cases, PSP was never raised in the differential diagnosis, and 4 of these patients were referred to the brain bank as part of studies on aging and dementia and had been evaluated by behavioral neurologists (eg, FLorida Autopsied Multi‐Ethnic cohort^10^). Given the retrospective nature of the study cohort, only cases with high‐quality antemortem medical documentation were included. To increase stringency, all cases with PSP‐FTD had a primary diagnosis or differential diagnosis of bvFTD made by at least 2 neurologists. For comparison of clinicopathological features, cases with PSP‐RS were selected based on quality and completeness of medical records; 31 cases met inclusions criteria, which also included matching for age, sex, and comorbid Alzheimer's‐type pathology with PSP‐FTD (Table 1). Clinical information (age at death, sex, symptoms at presentation, symptoms during the course of the illness, clinical diagnosis, disease duration, and family history) was obtained from available medical records.

**Table 1:** Demographics and neuropathology of PSP‐FTD and PSP‐RS

	PSP‐FTD	PSP‐RS
	(n = 15)	(n = 31)
Demographics		
Female	3 (20%)	10 (32%)
Age at death	73 (69, 75)	75 (72, 78)
Disease duration	9 (6, 10)	7 (6, 9)
Genetics		
MAPT (H1H1)	50%	73%
APOE (ε4 carrier)	33%	18%
Pathology		
Brain weight	1140 (1100, 1240)	1140 (1095, 1200)
Braak NFT stage	II (I‐II, III)	II‐III (I‐II, III)
Thal amyloid phase	0 (0, 3)	1 (0, 2)
Neurofibrillary tangle counts (X400), with thioflavin‐S fluorescent microscopy		
Middle frontal gyrus	0 (0, 0)	0 (0, 0)
Superior temporal gyrus	0 (0, 0)	0 (0, 0)
Inferior parietal lobule	0 (0, 0)	0 (0, 0)
Hippocampus — CA4	0 (0, 1)	0 (0, 1)
Hippocampus — CA2/3	0 (0, 1)	1(0, 2)
Hippocampus — CA1	1 (0, 2)	1 (0, 2)
Hippocampus — subiculum	1(0, 2.5)	1 (0, 2)
Neuronal loss (score with hematoxylin and eosin histochemistry)		
Substantia nigra	3 (2, 3)	2 (2, 3)
Locus coeruleus	3 (2.5, 3)	3 (2, 3)

Analyses used Kruskal‐Wallis analysis of variance on ranks, and data are displayed as median (25th percentile, 75th percentile) or percent of patients with the specific feature, unless otherwise noted. Genetic comparisons with Fisher's exact test. None of the variables differed significantly between the 2 groups.

### Clinical Assessment of PSP‐FTD and PSP‐RS

PSP‐FTD cases had clinical features of FTD described by Neary^11^ and were included if they had features consistent with the behavioral variant of FTD (bvFTD).^12^ Other frontotemporal syndromes such as progressive nonfluent aphasia, semantic dementia, or progressive asymmetrical rigidity and apraxia (ie, corticobasal syndrome) were excluded. The behavioral features recorded on each case were classified as: A, disinhibition; B, apathy; C, loss of sympathy or empathy; D, preservative, stereotyped, or compulsive/ritualistic behavior; and E, hyperorality and dietary changes. For clinical diagnosis of Richardson syndrome, we used criteria originally reported by Williams and coworkers, which are similar to recently published Movement Disorder Society criteria.^2^, ^13^

### Genetic Analyses

Genotyping was performed with a TaqMan allelic Discrimination Assay on an ABI 7900HT Fast Real‐Time PCR system (Applied Biosystems). One single‐nucleotide polymorphism (SNP; rs1052553) was used to determine the *MAPT* haplotype, and 2 SNPs (rs7412 and rs429358) were used to determine the *APOE* genotype.

### Microscopic Pathology

All cases had standardized dissections, sampling, and tissue processing, and they were all evaluated by a single neuropathologist (D.W.D.). Formalin‐fixed, paraffin‐embedded tissue samples were cut at a 5‐μm thickness and mounted on glass slides for study. In addition to histologic evaluation, the presence and severity of Alzheimer's pathology was assessed with thioflavin‐S fluorescent microscopy. A Braak neurofibrillary tangle stage^14^ and Thal amyloid phase^15^ were assigned to each case based on lesion counts in cortical and subcortical areas with thioflavin‐S fluorescent microscopy as previously described.^16^

### Immunohistochemistry

Immunohistochemistry was performed on 5‐μm‐thick sections of formalin‐fixed, paraffin embedded tissue. Glass‐mounted sections were deparaffinized in xylene and rehydrated in ethanol and distilled water. Immunohistochemistry for tau used an antibody to phospho‐serine 202 (CP13; mouse monoclonal; from Peter Davies, PhD, Feinstein Institute, North Shore Hospital, Manhasset, NY) and for TDP‐43, a rabbit polyclonal antibody to a mid‐region neoepitope (MC2085; from Leonard Petrucelli, PhD, Mayo Clinic Jacksonville, Jacksonville, FL). The following regions were evaluated with digital pathology: superior frontal gyrus, middle frontal gyrus, inferior temporal gyrus, and motor cortex. Immunohistochemical preparations were processed on a DAKO AutostainerPlus (Agilent/DAKO, Santa Clara, CA) with a DAKO EnVision+ system–HRP with 3,3′‐diaminobenzidine (DAB) as the chromogen. Nonspecific antibody binding was blocked with normal goat serum (Sigma, St. Louis, MO).

### Image Analysis

Digital microscopy methods have been previously described. Briefly, immunostained sections were scanned on an Aperio ScanScope XT slide scanner (Aperio Technologies, Vista, CA), producing a high‐resolution digital image. Digital image analysis was performed using Aperio ImageScope software. Several regions of interest were outlined from each image. A color deconvolution algorithm was used to count the number of pixels that were immunostained by the DAB chromogen in the manually outlined regions. The color channels for the color deconvolution algorithm were based on the DAB chromogen. Based on the chromogen staining density of the digital slide image, the algorithm assigns each pixel an intensity range, binned into strong staining (red), moderate staining (orange), weak staining (yellow), or no staining (blue). The signal for tau was considered positive if the pixel was red, orange, or yellow. Areas with no staining (white) and those below threshold (blue) were considered negative. The output variable was percentage of positive pixels relative to the total area of the region of interest.

### Statistical Analysis

Sigma Plot version 12 (Systat Software, San Jose, CA) was used for statistical analyses. Because of the small sample sizes, nonparametric Kruskal‐Wallis analysis of variance on ranks was performed on quantitative measures to assess differences in the median values. Post hoc pairwise comparisons were performed between each of the groups using the Mann‐Whitney rank sum test. For categorical data (eg, sex, *APOE* and *MAPT* genotypes, and clinical symptoms), a chi‐square test was used to compare group differences. Fisher's exact test was used for comparison of pairwise categorical data if the counts were less than 5. Correlative analysis was performed using Spearman rank order correlation. Kruskal‐Wallis rank sum tests were used for ordered categorical or continuous variables in pathogenic analyses. A statistically significant difference was considered for a 2‐sided *P* < 0.05. For comparison of tau pathology between PSP‐FTD and PSP‐RS, Mann‐Whitney rank sum tests were performed, and *P* < 0.0083 using a Bonferroni correction was considered significant.

## Results

### Demographics, Pathology, and Genetics

The 15 autopsy‐proven PSP cases with antemortem clinical diagnosis of bvFTD were compared with 31 cases of PSP with Richardson syndrome. By design, PSP‐FTD did not differ from PSP‐RS with respect to sex, age at death, disease duration, brain weight, Braak neurofibrillary tangle stage, or Thal amyloid phase. They were also similar in neurofibrillary tangle counts with thioflavin‐S fluorescent microscopy and the severity of neuronal loss assessed semiquantitatively in a ventrolateral cell group of the substantia nigra^17^ and in the locus coeruleus (Table 1). There were no differences in frequency of *MAPT* H1H1 or *APOE* ε4 between PSP‐FTD and PSP‐RS.

### Clinical Comparison of PSP‐FTD and PSP‐RS

Clinical features of PSP‐FTD and PSP‐RS at presentation and later in the disease course are summarized in Table 2. At initial presentation, only 1 patient with PSP‐FTD had unexplained falls (6%), whereas 94% of PSP‐RS patients had early unexplained falls or significant gait disturbance (*P* < 0.001). There was no difference in frequency of vertical gaze palsy or limb dysfunction. All PSP‐FTD patients had initial cognitive or behavioral problems, including personality changes (*P* < 0.001), a language disorder (*P* = 0.02), or memory complaints. These features were very uncommon as an initial complaint in PSP‐RS. Disinhibition was significantly more frequent in PSP‐FTD compared with PSP‐RS (*P* < 0.001), and a language disorder was also more frequent in PSP‐FTD (*P* = 0.008). In contrast, motor problems were universal in PSP‐RS patients at presentation. Parkinsonism, gait disorder, falls, and vertical gaze palsy were significantly more frequent during the disease course in PSP‐RS compared with PSP‐FTD. Although all PSP‐FTD cases were considered to have FTD, more than half of the PSP‐FTD patients also had mild motor problems, such as falls, gait problems, or parkinsonism (Table 2).

**Table 2:** Comparison of clinical features of PSP‐FTD and PSP‐RS

	PSP‐FTD	PSP‐RS	*P*
	(n = 15)	(n = 31)	
Initial presentation			
Motor dysfunction			
Parkinsonism	0 (0%)	4 (13%)	
Gait disorder and falls	1 (6%)	29 (94%)	<0.001
Vertical gaze palsy	0 (0%)	2 (6%)	
Limb dysfunction	0 (0%)	2 (6%)	
Cognitive impairment			
Personality or behavioral changes	10 (67%)	2 (6%)	<0.001
Language disorder	4 (27%)	1 (3%)	0.02
Memory problems	4 (24%)	2 (6%)	
Features during disease course			
Motor dysfunction			
Parkinsonism	2 (12%)	31 (100%)	<0.001
Gait disorder and falls	8 (53%)	31 (100%)	<0.001
Dystonia	0	1 (3%)	
Vertical gaze palsy	5 (33%)	31 (100%)	<0.001
Cognitive impairment			
Disinhibition	10 (67%)	3 (9%)	<0.001
Apathy or abulia	6 (40%)	5 (15%)	
Memory problems	3 (20%)	6 (18%)	
Language disorder	7 (41%)	3 (10%)	0.008
Limb apraxia	1 (7%)	2 (6%)	
Visuospatial disorder	2 (14%)	5 (16%)	

Data are displayed as frequency of a given clinical feature (percent of total in that group). Post hoc pairwise comparison analysis was performed with Mann‐Whitney rank sum test. Only statistically significant *P* values are shown.

### Pathological Findings

**Figure 1:**
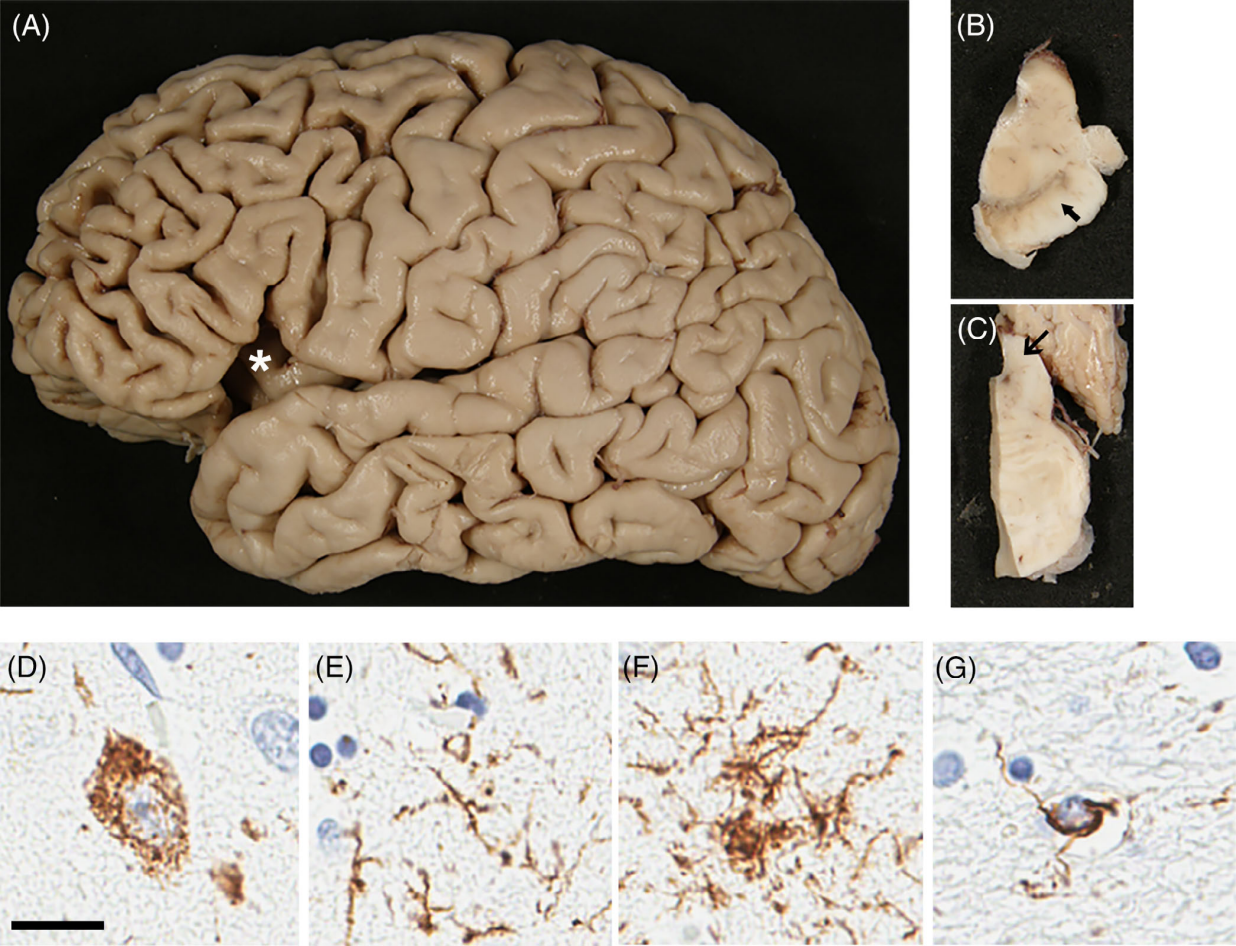
Macroscopic and phospho‐tau immunohistochemistry in PSP‐FTD. Cerebral atrophy is marked in frontal cortex (A). Pigmentation of substantia nigra is preserved (B).The superior cerebellar peduncle has no atrophy (C). Representative phospho‐tau immunohistochemistry of superior frontal cortex in PSP. Phospho‐tau pathology is present in both gray matter and white matter in PSP‐FTD and PSP‐RS. In gray matter, NFTs (D), threads (E), and tufted astrocytes (F) are illustrated, whereas coiled bodies are seen in the white matter (G). Scale bar: 20 μm

**Figure 2:**
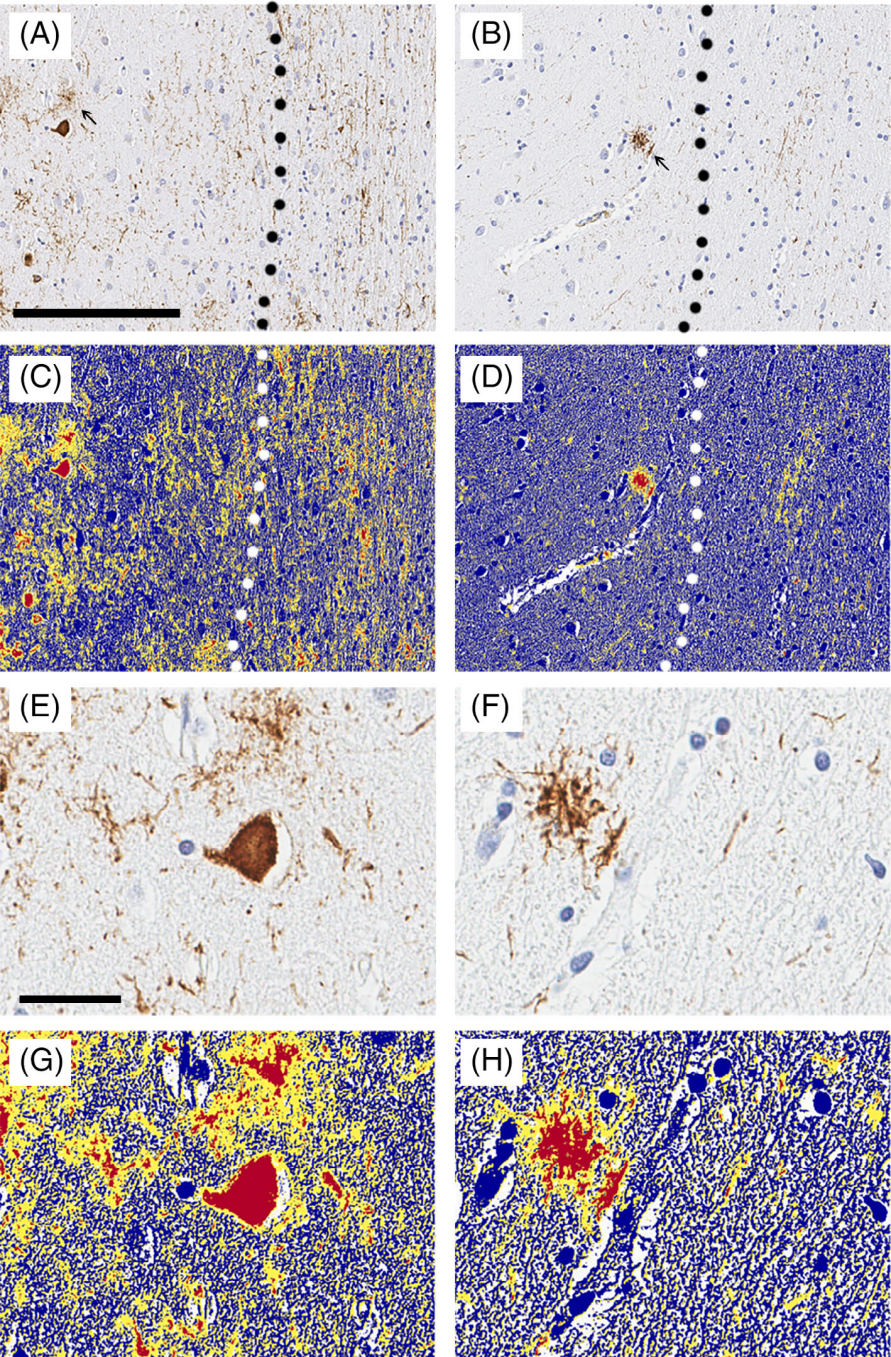
Image analysis of phospho‐tau in PSP‐FTD and PSP‐RS. Representative images of phospho‐tau immunohistochemistry in superior frontal cortex of PSP. Phospho‐tau pathology is present in both gray matter and white matter in PSP‐FTD (A), but much less in PSP‐RS (B). Tufted astrocytes are more frequent in PSP‐FTD than PSP‐RS (arrow). Dashed line indicates junction between gray matter and white matter. Scale bar: 200 μm. Digital image analysis of phospho‐tau immunohistochemistry in the superior frontal cortex of PSP‐FTD (C) and PSP‐RS (D). Higher magnification image of PSP‐FTD (E, G) and higher magnification image of digital analysis of PSP‐FTD (F, H). Application of the image analysis color deconvolution algorithm shows strong positive pixels as red. A positive pixel count algorithm was customized to quantify immunoreactive pixels (red), subtracting inverse pixels (blue), and background pixels (yellow). The analysis does not discriminate between NFTs, neuropil threads, coiled bodies, and tufted astrocytes. Dashed line indicates junction of gray matter and white matter. Scale bar: 150 μm (A–D). Scale bar: 30 μm (E–H).

Disproportionate atrophy was observed in the frontal lobe was noted. Pigment loss in the substantia nigra was not obvious, and the superior cerebellar peduncle had no significant atrophy in PSP‐FTD (Fig. 1A–C). Tau pathology was assessed in PSP‐FTD and PSP‐RS with phospho‐tau immunohistochemistry (Fig. 1D–G). Digital microscopy image analysis was used to quantify the density of phospho‐tau pathology (Fig. 2). In the superior frontal gyrus, PSP‐FTD patientshad significantly greater phospho‐tau burden compared with PSP‐RS (*P* = 0.008). PSP‐FTD patients also had greater tau pathology in the white matter of the superior frontal gyrus (*P* = 0.08), middle frontal gyrus (*P* = 0.07), and inferior temporal gyrus, reaching statistical significance in the inferior temporal gyrus (*P* = 0.001); see Table 3. These results suggest that tau density and distribution might be associated with behavioral features in PSP‐FTD.

**Table 3:** Summary of neuropathological comparison between PSP‐FTD and PSP‐RS

	PSP‐FTD	PSP‐RS	*P*
	(n = 15)	(n = 31)	
Tau pathology (lesion scores)			
Neuronal tangles and pretangles			
Superior frontal gyrus	2.0 (2.0, 3.0)	2.0 (1.0, 2.0)	0.001
Middle frontal gyrus	2.0 (1.0, 2.0)	2.0 (1.0, 2.3)	
Inferior temporal gyrus	1.0 (1.0, 2.0)	1.0 (0.0, 1.0)	0.008
Neuropil threads			
Superior frontal gyrus	2.0 (1.0, 2.0)	1.0 (0.0, 1.3)	0.04
Middle frontal gyrus	1.0 (1.0, 2.0)	1.0 (0.0, 2.0)	
Inferior temporal gyrus	0.0 (0.0, 2.0)	0.0 (0.0, 0.0)	
Oligodendroglial coiled bodies			
Superior frontal gyrus	2.0 (1.0, 2.0)	1.0 (1.0, 2.0)	0.04
Middle frontal gyrus	2.0 (2.0, 3.0)	2.0 (1.0, 2.3)	0.03
Inferior temporal gyrus	0.0 (0.0, 1.0)	0.0 (0.0, 1.0)	
Tau burden (image analysis)			
Cortical gray matter			
Superior frontal gyrus	0.6 (0.3, 1.2)	0.3 (0.15, 0.49)	0.008
Middle frontal gyrus	0.3 (0.2, 0.4)	0.2 (0.08, 0.49)	
Inferior temporal gyrus	0.1 (0.1, 0.4)	0.1 (0.0, 0.2)	
Subcortical white mater			
Superior frontal gyrus	0.4 (0.2, 0.8)	0.2 (0.1, 0.4)	
Middle frontal gyrus	0.2 (0.1, 0.4)	0.1 (0.08, 0.3)	
Inferior temporal gyrus	0.1 (0.1, 0.3)	0.04 (0.0, 0.1)	0.001

All variables were analyzed with Kruskal‐Wallis analysis of variance on ranks, and data are displayed as median (25th percentile, 75th percentile), unless otherwise noted. Post hoc pairwise comparison analysis was performed with Mann‐Whitney rank sum test. Only statistically significant *P* values are shown. Corpus callosum thickness measures are in centimeters.

We also evaluated the nature of tau pathologies, focusing on neuronal pathology (neurofibrillary tangles [NFTs] and pretangles), neuropil threads, and glial lesions (tufted astrocytes and oligodendroglial coiled bodies). Neuronal tau pathology was significantly greater in the superior frontal gyrus and inferior temporal gyrus of PSP‐FTD compared with PSP‐RS (Table 3). Neuropil threads were abundant in both PSP‐FTD and PSP‐RS, with significantly more threads in the superior frontal gyrus and motor cortex in PSP‐FTD compared with PSP‐RS (Table 3). Tufted astrocytes did not differ between PSP‐FTD and PSP‐RS (not shown). Oligodendroglial coiled bodies were more numerous in the superior frontal gyrus and middle frontal gyrus of PSP‐FTD compared with PSP‐RS (Table 3). These results suggest that greater neuronal and glial tau pathology in both gray matter and white matter of the frontal and temporal lobes is associated with PSP‐FTD.

## Discussion

PSP‐RS is a distinctive disorder with high diagnostic accuracy confirmed by postmortem neuropathologic studies.^9^ Early clinical manifestations of PSP‐RS are related to motor problems, with frequent falls and vertical gaze palsy^18^; however, many patients also develop cognitive dysfunction that has characteristics of a subcortical dementia, characterized by cognitive dysfunction, bradyphrenia, slow responses, and poor recall.^19^ A subset of PSP patients has more marked cognitive deficits and features that mimic bvFTD.^20^, ^21^ In this study, we aimed to better understand clinical and pathologic features of PSP‐FTD. In the Mayo Clinic brain bank for neurodegenerative disorders, we identified 15 patients with pathologically confirmed PSP and antemortem clinical features of bvFTD (PSP‐FTD). Only 1 patient had early unexplained falls typical of PSP‐RS, whereas the remaining patients had cognitive and behavioral features that overshadowed motor features. Most of the PSP‐FTD patients developed motor dysfunction during the disease course, but 4 patients never had significant motor problems. These 4 patients were thought to have bvFTD as the final diagnosis before death. All PSP‐FTD patients had features consistent with bvFTD. The most common behavioral problem was that of disinhibition. Abulic or apathetic features are common in subcortical dementia,^19^ but like our series, Gerstenecker and coworkers also reported that only 8% of a series of 154 clinically diagnosed PSP patients had moderate to severe apathy, whereas disinhibition was present in 21%.^22^

Clinical features are known to overlap and to evolve with disease progression in individuals with frontotemporal degenerative disorders.^23^ In a series of 60 autopsy‐confirmed cases of frontotemporal degeneration studied by Kertesz and coworkers, more than half (n = 32) presented with bvFTD. In none of the cases was PSP found at autopsy. The most common tauopathies associated with bvFTD were corticobasal degeneration and Pick's disease.^23^ Review of the literature would also suggest that bvFTD is an uncommon presentation of PSP. In a study of 66 autopsy‐confirmed cases of PSP by Hassan and coworkers, only 3 cases of 66 PSP patients (4%) presented with behavioral and personality changes consistent with bvFTD.^4^ A retrospective multicenter European study of autopsy‐confirmed (“definite”) PSP showed the frequency of FTD‐like features in 12 of 100 cases.^5^ In that series, 7 patients had both progressive nonfluent aphasia and bvFTD, whereas 4 had only bvFTD. Bigio and coworkers studied tau pathology in the frontal neocortex (both gray matter and white matter) of a series of typical PSP patients and PSP patients with dementia. The dementia characteristics were not specifically those of bvFTD, but rather Alzheimer's‐type dementia with or without parkinsonism, hydrocephalus, Binswanger's disease, or corticobasal syndrome. Like the current study, they found increased cortical tau pathology in PSP with dementia compared with PSP without dementia.^24^ In that series, most patients presented with attention and memory deficits, with fewer showing language problems; only 2 patients had early behavioral problems.^24^ Although these previous studies had investigated the neuropathology of PSP with dementia, the current study is the first to focus exclusively on patients meeting clinical criteria for bvFTD.

A more significant body of literature has characterized neuroimaging features of PSP presenting with frontal executive and behavioral dysfunction.^25^, ^26^, ^27^ Cordato and coworkers correlated behavioral changes in PSP with atrophy in the orbitofrontal cortex and the midbrain.^26^ Using voxel‐based morphometry, Josephs and coworkers reported that behavioral changes in PSP correlated with frontostriatal volume loss.^25^ Chiu and coworkers, comparing 21 patients with PSP‐RS with 11 patients with PSP‐FTD, reported that frontobehavioral features in PSP were associated with hypoperfusion in the posterior part of the cingulate gyrus and that hypoperfusion correlated with the severity of executive dysfunction.^28^ The neuropathologic basis for these neuroimaging observations was not reported. In the present study, we found that PSP‐FTD had significantly greater total tau burden in the superior frontal gyrus in both cortical gray matter and subcortical white matter, as well as greater white matter tau pathology in middle frontal and inferior temporal gyri. The lesion types most likely to account for this increased burden were pretangles and neuropil threads, but oligodendroglial coiled bodies were also increased in PSP‐FTD compared with PSP‐RS. Our results provide evidence that not only total tau burden, but also neuronal and oligodendroglial tau pathology is associated with bvFTD presentation of PSP.

Regarding white matter pathology in PSP and specifically in PSP‐FTD, genome‐wide association studies of PSP and CBD identified a variant near *MOBP*, a myelin oligodendrocyte protein.^29^, ^30^ It is of note that neuroimaging studies have suggested that white‐matter abnormalities may correlate with apathy and impulsivity in PSP.^31^ Caso and coworkers reported that white‐matter damage showed significant correlation with cognitive features in PSP‐RS, but regional cortical thinning did not.^32^ In this study, we observed significantly greater density of tau pathology in both gray and white matter of PSP‐FTD.

The present study has strengths and limitations. Neuropathologic characterization is a strength, in that all cases were referred to a single brain bank, and neuropathologic procedures were standardized. The analyses included both semiquantitative lesion burden scores and tau burden using digital pathology and image analysis in regions of interest. A weakness is that the PSP cases were a sample of convenience and were not necessarily representative of a population‐based cohort. Patients with atypical clinical presentations may disproportionately come to autopsy,^33^ which might imply that the bvFTD subtype of PSP may be even less frequent than that observed (ie, less than 2%). Another weakness is that medical documentation was variable and not standardized; however, only cases with high‐quality medical documentation and confirmation of the diagnosis by 2 neurologists were included in the final analyses.

In conclusion, we found that PSP‐FTD represents an uncommon clinical subtype of PSP, with disinhibition more frequent than apathy/abulia. PSP‐FTD differs from PSP‐RS by greater tau pathology in the gray and white matter of the superior frontal gyrus, middle frontal gyrus, and inferior temporal gyrus. PSP‐FTD does not differ from PSP‐RS with respect to common genetic risk factors (eg, *APOE* and *MAPT*). Further studies are warranted to understand the factors that contribute to this unusual presentation of PSP.
